# The nature and nurture of primary and secondary callous–unemotional traits: evidence from two independent twin samples

**DOI:** 10.1111/jcpp.70107

**Published:** 2026-01-05

**Authors:** Rachel C. Tomlinson, Patrizia Pezzoli, Essi Viding, Stephane A. De Brito, Kelly L. Klump, S. Alexandra Burt, Luke W. Hyde

**Affiliations:** ^1^ Department of Psychology University of Michigan Ann Arbor MI USA; ^2^ Department of Epidemiology and Biostatistics University of California San Francisco San Francisco CA USA; ^3^ Division of Psychology and Language Sciences University College London London UK; ^4^ School of Psychology University of Birmingham Birmingham UK; ^5^ Department of Psychology Michigan State University East Lansing MI USA

**Keywords:** Callous–unemotional traits, anxiety, behavior genetics, twin study, genotype‐environment interaction

## Abstract

**Background:**

Callous–unemotional (CU) traits identify youth with more severe and chronic trajectories of conduct problems. However, the etiology of CU traits may be heterogeneous, undermining the search for effective treatments. The level of co‐occurring anxiety has been used to identify “primary” (lower anxiety) versus “secondary” (higher anxiety) variants of CU traits. The primary variant has been hypothesized to emerge from strong genetic influence and secondary variants as an adaptation to adversity, such as exposure to childhood maltreatment. However, little research has tested this hypothesis directly.

**Methods:**

We examined whether anxiety moderates the etiology of CU traits to determine whether this phenotypic feature can help distinguish CU traits with stronger genetic or environmental risk. In two population‐based twin cohorts (initial sample: *N* = 1,196, aged 6–11, oversampled for exposure to neighborhood disadvantage; follow‐up sample: *N* = 13,486, age 7), we used genotype‐by‐environment interaction twin modeling to examine if parent‐reported child anxiety moderated the etiology of concurrent parent‐reported child CU traits.

**Results:**

Anxiety moderated the etiology of CU traits across both samples, such that nonshared environmental influences increased as anxiety increased. Additionally, in the larger sample, genetic influences decreased with increasing anxiety.

**Conclusions:**

These findings support theories suggesting that co‐occurring anxiety may distinguish CU traits with different origins: CU traits with higher anxiety appear more influenced by nonshared environmental factors—potentially including adversity—and may show weaker genetic influence. Assessing for co‐occurring child anxiety is likely important for diagnosing and personalizing treatments among children with CU traits.

## Introduction

Conduct problems that start in childhood are concurrently and prospectively associated with substantial personal, societal, and economic costs across the lifespan (Fairchild et al., 2019). Childhood conduct problems not only predict adolescent conduct disorder and adult antisocial personality disorder but also a number of other poor mental health, physical health, educational, and employment outcomes (Fairchild et al., 2019). Consequently, understanding, preventing, and treating childhood conduct problems is a major public health priority.

Key to preventing these costly outcomes is identifying children who will have more chronic trajectories of antisocial behavior. Callous–unemotional (CU) traits, defined by low empathy, remorselessness, and shallow affect—symptoms also seen in adult psychopathy—have been added as a diagnostic specifier to identify a group of children with conduct disorder and more severe trajectories of behavior problems (“with limited prosocial emotions” specifier in DSM‐5 and ICD‐11; American Psychiatric Association, 2013; World Health Organization, 2022). In ICD‐11, this specifier is also applied to oppositional defiant disorder, underscoring recognition that CU traits are clinically relevant across multiple childhood disruptive behavior disorders (World Health Organization, 2022). The inclusion of this specifier was informed by decades of empirical research suggesting that children with conduct disorder and high levels of CU traits can be distinguished from their peers with low levels of CU traits at the etiological, neural, cognitive, and behavioral level (Frick, Ray, Thornton, & Kahn, 2014; Viding, McCrory, & Seara‐Cardoso, 2014). Moreover, although children with conduct problems and high levels of CU traits do benefit from standard treatments, such as parent training and emotion recognition training, these treatments do not bring their behavior into normative range (Perlstein, Fair, Hong, & Waller, 2023; Wilkinson, Waller, & Viding, 2015). These outcomes are in line with their more severe behavioral profile and their neurocognitive presentation that may not be fully addressed in standard treatments. Indeed, adjunct protocols that address the particular presentation of children with CU traits can result in more robust treatment gains for this group (Fleming, Neo, Kaouar, & Kimonis, 2023).

Further advancement in personalization of treatment is thought to be complicated by the fact that CU traits themselves may develop via different pathways (Viding & Kimonis, 2018). Early research on adult psychopathy and child CU traits suggested that callous lack of empathy is not typically accompanied by anxiety or internalizing problems (Frick, Lilienfeld, Ellis, Loney, & Silverthorn, 1999; Pardini & Fite, 2010). Yet accumulating evidence from more recent studies suggests that callousness may co‐occur with high levels of anxiety in some children with CU traits and in some adults with psychopathy (Fanti, Demetriou, & Kimonis, 2013; Kahn et al., 2013; Sethi et al., 2018). Such individuals have been described as presenting with “secondary” variants of CU traits (or psychopathy in adults), in contrast with the “primary” variants that occur in the absence of clinically significant anxiety (Kimonis, 2023). This “secondary” presentation has been linked to experiences of significant childhood adversity and trauma (Kimonis, Fanti, Isoma, & Donoghue, 2013; Todorov et al., 2024), and is proposed to have a distinct etiology from the primary, putatively more heritable, presentation (Viding & Kimonis, 2018). “Secondary” CU traits have therefore been suggested to represent a “behavioral phenocopy” of primary CU traits—arising as an adaptation to environmental adversity (Viding & Kimonis, 2018). Evidence also suggests that these variants may differ in their responsiveness to treatment: Although both groups can benefit from parent training adapted for CU traits, in one trial children with secondary CU traits showed faster improvements during treatment but a quicker deterioration in gains during follow‐up, highlighting the need for further treatment tailoring across groups (Fleming et al., 2023). Understanding variant‐specific etiological pathways is critical for such tailoring. If secondary CU traits arise as an adaptation to adversity, treatment may need to target specific environmental factors and co‐occurring anxiety. Conversely, primary CU traits may benefit more from interventions targeting temperamental factors such as socioemotional insensitivity (Fleming et al., 2023).

Across multiple studies, CU traits have been shown to be moderately to strongly heritable when measured in middle childhood and adolescence (36%–67%) with the remaining variability largely attributed to nonshared environmental influences (Moore, Blair, Hettema, & Roberson‐Nay, 2019). However, studies have also shown that factors like parenting can moderate the heritability of CU traits (Henry et al., 2018; Tomlinson, Hyde, Dotterer, Klump, & Burt, 2022), indicating that the etiology of CU traits can vary as a function of life experiences. Given that “secondary” CU traits (i.e., CU traits with high anxiety) are linked to early adversity and trauma (Craig, Goulter, & Moretti, 2020), CU traits are hypothesized to be less heritable and more strongly influenced by environmental factors at higher levels of anxiety. Only one study, to our knowledge, has investigated whether those with low vs. higher levels of anxiety have a different etiology to their CU traits. Using data from a population‐based cohort in the UK — the Twins Early Development Study (TEDS; Humayun, Kahn, Frick, & Viding, 2014; Lockhart et al., 2023; Oliver & Plomin, 2012; Rimfeld et al., 2019) — Humayun et al. (2014) used DeFries‐Fulker extremes analysis to estimate the genetic and shared environmental influences on CU traits among 7‐year‐old same‐sex twins with high levels of CU traits (top 10% of CU traits), dividing them into high‐ and low‐anxiety groups based on teacher reports. The study found strong genetic influences on CU traits in the high‐CU traits, low‐anxiety group (*n* = 627; *h* ^2^ = 0.75). The genetic influences on CU traits in the high‐CU high‐anxiety group (*n* = 119; *h* ^2^ = 0.66) were slightly lower, but also strong and not statistically different from those observed for the low‐anxiety group. No shared environmental influences were observed for either group. As such, the remaining variance in CU traits in both groups was attributable to nonshared environmental influences and error variance (0.25–0.34), with no evidence that these effects differed significantly between variants. Although this study did not report a statistically significant etiological difference between the groups, it is difficult to draw strong conclusions from this study due to the small size of the high‐anxiety group (*n* = 119), and given that extremes were drawn from a population‐based rather than high‐risk or clinical cohort. The hypothesized effects may be more apparent with more powerful methodology in well‐powered or high‐risk samples.

Here, we examined the etiology of CU traits in middle childhood, as a function of anxiety levels, in two complementary population‐based samples. To rigorously evaluate whether anxiety moderates the etiology of CU traits, we applied the extended univariate G×E modeling approach, which represents a significant methodological advancement over previous work, allowing us to examine genetic and environmental influences across the entire spectrum of CU traits and anxiety and to test directly whether anxiety moderates these influences. The first sample was a cohort in the USA with oversampling for families living in lower‐income neighborhoods, which has been shown to increase risk for antisocial behavior, particularly in youth with high levels of CU traits (Markowitz, Ryan, & Marsh, 2014). Next, we conducted a preregistered conceptual replication in the TEDS cohort from the UK, using identical analytic approaches, but with a substantially larger sample (*N* = 13,486) than both the previous study in TEDS (N = 1,202; Humayun et al., 2014) and our first at‐risk sample (*N* = 1,196), improving power to detect moderation effects. We predicted that anxiety would moderate the etiology of CU traits, with environmental influences increasing and genetic influences decreasing as anxiety increases.

## Methods

### Participants

#### TBED‐C

The initial sample included data from families assessed as part of the Twin Study of Behavioral and Emotional Development in Children (TBED‐C; Burt & Klump, 2019), a project within the Michigan State University Twin Registry. TBED‐C families were identified through birth records, and participated in their intake assessment between 2008 and 2015. The 598 twin pairs (231 MZ, 367 DZ) included in the present study were additionally required to reside in a neighborhood with above‐average levels of poverty. The present study only included families for whom CU traits data were collected (the measure was added partway through the multiyear study). The twins were 6 to 11 years old (Mean = 8.07 years, SD = 1.53 years; 50.7% male). Compared to the full eligible sample, children with CU data were slightly younger than those without (Mean = 8.07 vs. 8.36 years, *p* < 0.01), but did not differ by sex (*p* = 0.50) or anxiety symptoms (*p* = 0.12). The breakdown of twins' parent‐reported ethnicity reflected the surrounding recruitment area (81% White, 10% African‐American, 6% Other, 1% Native American, 1% Latino/Latina, <1% Asian). Zygosity was established using physical similarity questionnaires (administered to the twins and/or their parents) that show accuracies of 95% or better (Peeters, Van Gestel, Vlietinck, Derom, & Derom, 1998). Discrepancies were resolved through review of zygosity items or by DNA markers.

#### TEDS

The follow‐up sample included participants from TEDS, a longitudinal cohort of twins born in England and Wales between 1994 and 1996 (Lockhart et al., 2023; Oliver & Plomin, 2012; Rimfeld et al., 2019). All twins born in England and Wales during the study period were invited to participate by the UK Office for National Statistics (for additional details on recruitment and representativeness of the UK population see Lockhart et al., 2023; Rimfeld et al., 2019). We analyzed data collected when twins were 7 years old. The present study only included twins with data on CU traits and anxiety and excluded twins with serious medical or perinatal conditions or missing zygosity information. The final sample included 4,888 MZ twins (2,294 males, 2,594 females) and 8,598 DZ twins, including 4,344 same‐sex pairs (2,112 males, 2,232 females) and 4,254 opposite‐sex pairs. Most participants (93.90%) identified as White. Zygosity was determined using a parent‐rated questionnaire on physical similarity and confirmed using DNA marker testing in most cases (Freeman et al., 2003). Children with CU data did not differ from the full eligible sample in age (*p* = 0.23), sex (*p* = 0.46), or anxiety symptoms (*p* = 0.57).

### Measures

#### Callous–unemotional (CU) traits

##### TBED‐C

CU traits were assessed via mother report on the 24 item Inventory of Callous‐Unemotional Traits (ICU; Essau, Sasagawa, & Frick, 2006) which includes three subscales: callousness (e.g., “Does not care who gets hurt to get what s/he wants”), uncaring (e.g., “Cares about how well s/he does at school or work”; reversed), and unemotional (e.g., “Does not show his/her emotions to others”) traits. The ICU has shown acceptable internal consistency for the total score across multiple studies (Cardinale & Marsh, 2020) and higher scores predict differential developmental trajectories for children with conduct problems (Frick et al., 2014). Aligned with prior factor analytic work of the parent‐report ICU (Waller et al., 2015) and consistent with prior studies in this cohort (Tomlinson et al., 2022), we utilized a 22‐item total score, excluding items 10 and 23 (*α* = 0.78, ω = 0.92). As a sensitivity check, we also evaluated models using the full ICU sum score; results were consistent, and we therefore retained the 22‐item score as our primary measure.

##### TEDS

CU traits were assessed using four items from the Strengths and Difficulties Questionnaire (Goodman, 2006) and three items from the CU subscale of the Antisocial Process Screening Device (Frick & Hare, 2002). Questionnaires for each twin were completed by parents, predominantly biological mothers. This combination of SDQ and APSD items is consistent with prior measurement of CU traits in the TEDS sample (Viding, Blair, Moffitt, & Plomin, 2005), and also aligned with the University of New South Wales (UNSW) approach to assessing CU traits (Dadds, Fraser, Frost, & Hawes, 2005). It was chosen by the TEDS team to represent key features of CU traits and has been previously validated in TEDS (Pezzoli et al., 2024). Consistent with TBED‐C, we created sum scores for analysis (*α* = 0.56, *ω* = 0.64).

#### Anxiety

##### TBED‐C

Anxiety symptoms were assessed via a sum score of mother report of anxiety via the DSM‐Oriented Anxiety Problems subscale on the Child Behavior Checklist (CBCL; Achenbach & Rescorla, 2001; *α* = 0.70, *ω* = 0.87). The DSM‐Oriented scales of the CBCL are widely used and have demonstrated excellent reliability and construct validity (Nakamura, Ebesutani, Bernstein, & Chorpita, 2008).

##### TEDS

Anxiety was assessed using a sum score of mother report on the Anxiety‐Related Behaviors Questionnaire (ARBQ), which measures the domains of negative affect, negative cognition, fear, and social anxiety and has previously demonstrated high internal consistency and good construct validity (Eley et al., 2003; *α* = 0.74, *ω* = 0.78).

### Analyses

We first completed analyses in TBED‐C, a population‐based sample with enrichment for risk. We then preregistered and completed a follow‐up analysis in the TEDS sample (https://osf.io/wt85h) as a conceptual replication and to extend findings to a larger, nonenriched sample.

Twin analyses leverage the difference in the proportion of genes shared between monozygotic (MZ) twins (who share 100% of their segregating genes) and dizygotic (DZ) twins (who share roughly 50% of their segregating genes) to estimate additive genetic (A), shared environmental (C), and nonshared environmental (E) contributions to a given phenotype (see Plomin, DeFries, Knopik, & Neiderhiser, 2012). Before performing model fitting, we calculated intraclass twin correlations (ICCs) using the double‐entry method, which removes variance due to twin ordering within each pair (Knopik, Neiderhiser, DeFries, & Plomin, 2017).

We first evaluated the etiology of CU traits using a univariate ACE model. We also tested an alternate, AE model, in which C is set to 0, to assess whether shared environmental influences were necessary to explain the variance in CU traits. As child age and sex correlate with CU traits (Essau et al., 2006) and anxiety (Avenevoli, 2012), we regressed out age and sex effects from both variables before analyses (i.e., used the residuals from regressions with age and sex predicting CU traits and anxiety). Additionally, it is generally recommended that unstandardized or absolute ACE estimates be presented for the G×E interaction models described below (Purcell, 2002), thus we used the standardized residual from this regression as our CU traits score (Mean = 0, SD = 1) to facilitate interpretation. To confirm our conservative approach did not introduce spurious effects, we conducted sensitivity analyses without regressing out age and sex from CU traits and anxiety.

**Figure 1:**
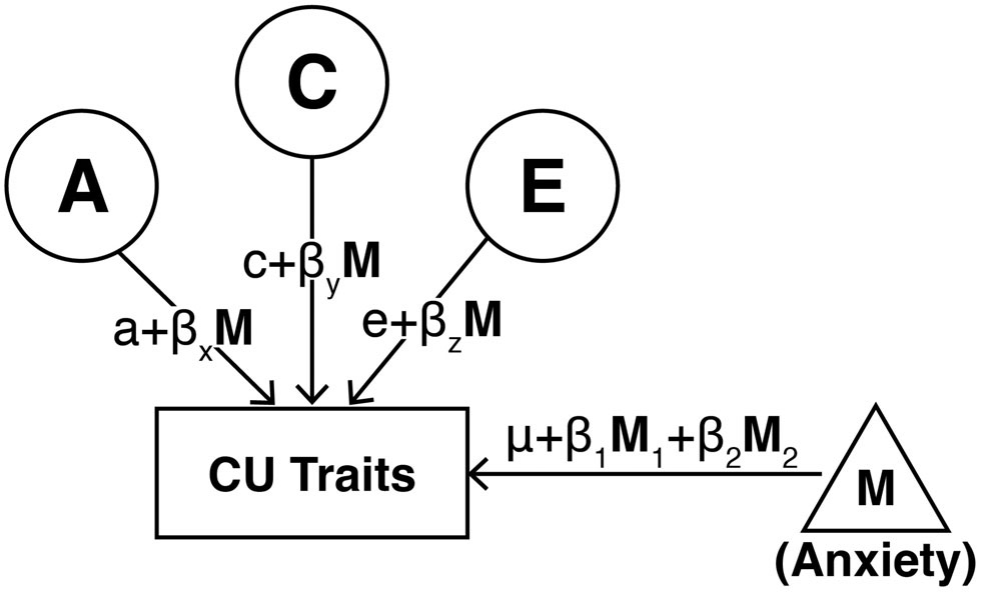
Extended univariate gene–environment interaction (G×E) model. This figure depicts the full extended univariate gene–environment (G×E) model (van der Sluis et al., 2012) for one twin. A, C, and E represent genetic, shared environmental, and nonshared environmental influences, respectively. M represents the moderator of interest. In this model, the variance decomposition (e.g., A, C, and E) was modeled as a function of the moderator (M). To account for gene–environment correlation (rGE), moderator values of both twins were entered in a means model of each twin's CU traits, and moderation was modeled on the variance in CU traits which did not overlap with the moderator. For ease of presentation, the co‐twin variables and paths are not shown here, though these were estimated in the model

We then evaluated whether anxiety moderated the etiology of CU traits using the “extended univariate G×E model” (Figure 1; Purcell, 2002; van der Sluis, Posthuma, & Dolan, 2012). In this model, the variance decomposition of CU traits was modeled as a function of anxiety, which we rescaled from 0 to 1 by flooring and dividing by its maximum. Notably, the extended univariate G×E model allows twins to differ from each other on the moderator variable. To address potential confounding due to gene–environment correlation (e.g., twins with higher genetic predisposition to CU traits contributing to the generation of anxiety‐provoking environments), we included both each twin's own anxiety score and their co‐twin's anxiety score in the means model of CU traits. This approach removes variance in CU traits that is correlated with either twin's anxiety, leaving the moderation to operate on variance unique to CU traits (i.e., that which does not overlap with anxiety) and thereby reducing bias due to rGE (van der Sluis et al., 2012). The initial, least restrictive model allowed for independent linear moderation of A, C, and E. We then fit a more restrictive model which constrained C and the associated linear moderation to be 0 and evaluated the change in model fit. As recommended by van der Sluis et al. (2012), we also ran a bivariate G×E model to confirm that the etiologic moderation we observed was indeed present on the variance that is unique to CU traits. This more computationally intensive model calculates variance component estimates for both the variable of interest and the moderator variable, as well as the overlap between the two. We also conducted a post hoc sensitivity analysis within TBED‐C in which externalizing symptoms, rather than CU traits, were specified as the variable of interest, to test whether any observed moderation was specific to CU traits or reflected externalizing more broadly.

Analyses were conducted using Mplus version 8.20 (Muthén & Muthén, 1998–2017) and R version 4.3.0, packages TwinAnalysis (Voronin, 2022) and MplusAutomation (Hallquist & Wiley, 2018). We fit models using full‐information maximum‐likelihood techniques. We evaluated model fit for the G×E models using two indices that balance overall fit with model parsimony: the Akaike's Information Criterion (AIC) and the Bayesian Information Criterion (BIC), with lower values indicating better fit. When available, we also considered root mean square of approximation (RMSEA) for which a lower value indicates better fit. For hypothesis testing, we adopted the traditional significance level of *p* < 0.05.

## Results

See Table S1 for descriptive statistics in the full samples and stratified by sex. Phenotypic correlations between anxiety and CU traits were small and positive within the TBED‐C sample (*r* = 0.17, *p* < 0.01), and very small and negative within the TEDS sample (*r* = −0.03, *p* = 0.04). Within TBED‐C, ICCs for CU traits were 0.46 (MZ) and 0.20 (DZ), and ICCs for anxiety were 0.42 (MZ) and 0.40 (DZ). Within TEDS, ICCs for CU traits were 0.65 (MZ) and 0.37 (DZ) twins, whereas for anxiety, ICCs were 0.69 (MZ) and 0.44 (DZ; see Table S2 for cross‐trait cross‐twin correlations).

### Univariate models

#### TBED‐C

Within TBED‐C, we found no evidence for shared environmental (C) influences on CU traits (Table S3). The best‐fitting univariate model was the AE model that estimated genetic influences (A) at 0.51 (95% CI [0.37, 0.60]) and nonshared environmental influences (E) at 0.49 (95% CI [0.40, 0.59]), indicative of moderate genetic and nonshared environmental influences.

#### TEDS

Within TEDS, we estimated genetic influences (A) at 0.59 (95% CI [0.55, 0.64]), shared environmental influences (C) at 0.07 (95% CI [0.03, 0.10]), and nonshared environmental influences (E) at 0.34 (95% CI [0.33, 0.35]). Considering the very small C estimate, for consistency with the TBED‐C analyses, we also tested an AE model. The AE model also fit the data (Table S3) and estimated genetic influences (A) at 0.67 (95% CI [0.65, 0.68]) and nonshared environmental influences (E) at 0.33 (95% CI [0.32, 0.35]), indicative of strong genetic and moderate nonshared environmental influences.

### G×E models

#### TBED‐C

**Figure 2:**
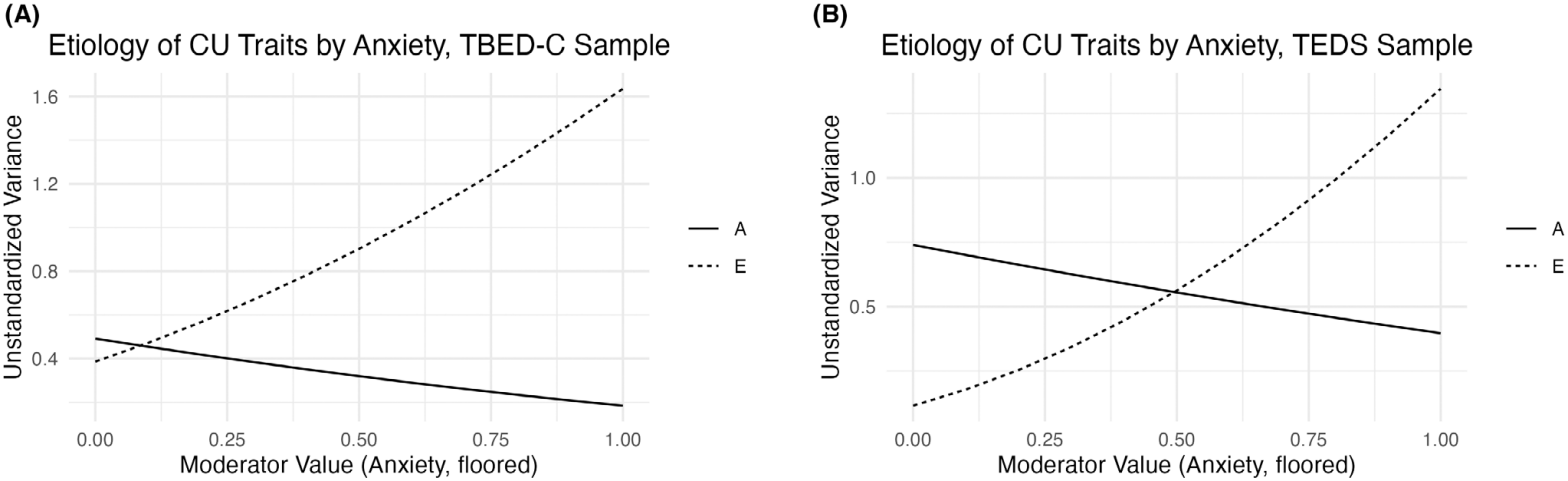
Child anxiety moderates the etiology of callous–unemotional (CU) traits. This figure depicts unstandardized additive genetic (A) and nonshared environmental (E) contributions to CU traits as predicted by the best‐fitting genotype × environment (G×E) interaction models at varying levels of parent‐reported child anxiety problems within two samples, TBED‐C (A; *N* = 595 pairs, 230 MZ) and TEDS (B; *N* = 6,743 pairs, 2,444 MZ). Within both samples, nonshared environmental influences on CU traits increased with increasing anxiety (*p* < 0.01). Additionally, within TEDS, the heritability of CU traits decreased with increasing anxiety (*p* < 0.01)

Consistent with univariate results, the best‐fitting extended univariate G×E model was the AE model with AE moderation (i.e., shared environmental influences (C) set to 0; Table 1; Figure 2). The model indicated increasing nonshared environmental influences on CU traits with increasing anxiety (E1 = 0.66, *p* < 0.01; Figure 2). Though A moderation was included in the model, it was not significant (A1 = −0.27, *p* = 0.45; Figure 2). To confirm that the etiologic moderation we observed was present for the variance unique to CU traits (van der Sluis et al., 2012), we also ran the bivariate G×E model (Purcell, 2002). Results indicated that the etiologic moderation in question was indeed specific to the unique path, though the sample was underpowered for this complex model (E1 unique = 0.54, *p* = 0.18; E1 shared = −0.06, *p* = 0.83).

**Table 1:** Genotype × Environment interaction models for TBED‐C and TEDS samples

Model	A	A1	C	C1	E	E1	AIC	BIC
*TBED‐C*								
ACE, ACE moderation	0.67*** [0.46, 0.88]	−0.12 [−1.15, 0.92]	−0.21 [−0.72, 0.30]	0.72 [−1.00, 2.44]	0.63*** [0.53, 0.72]	0.59 [0.00, 1.08]	3254.82	3313.94
**AE**, **AE moderation**	0.70*** **[0.59, 0.81]**	**−0.27** **[−0.97, 0.43]**			0.62*** **[0.53, 0.70]**	0.66** **[0.18, 1.13]**	**3251.00**	**3300.26**
*TEDS*								
**ACE**, **ACE moderation**	0.56*** **[0.50, 0.62]**	0.63*** **[0.46, 0.81]**	0.80*** **[0.74, 0.85]**	−1.83*** **[−2.03, −1.64]**	0.38*** **[0.35, 0.41]**	0.64*** **[0.53, 0.75]**	**35747.78**	**35829.58**
ACE, AE moderation	0.80*** [0.76, 0.84]	−0.33** [−0.49, −0.17]	0.37*** [0.31, 0.43]		0.34*** [0.31, 0.36]	0.94*** [0.84, 1.05]	35856.133	35931.112
AE, AE moderation	0.87*** [0.84, 0.90]	−0.22** [−0.34, −0.10]			0.34*** [0.32, 0.36]	0.87*** [0.77, 0.97]	35879.16	35947.32

This table depicts unstandardized estimates and model fit statistics for the extended univariate genotype × environment (G×E) interaction models. *N* = 595 pairs (230 monozygotic) for TBED‐C, *N* = 6,743 pairs (2,444 MZ) for TEDS. The first model listed for each moderator is the full ACE model of callous‐unemotional traits by anxiety, with linear moderation (A1, C1, E1) allowed on A, C, and E terms. The best‐fitting models are indicated in bold. In the TEDS sample, while the ACE moderation model fit the data best, the AE moderation model also demonstrated a strong fit (not statistically significantly worse fit than the ACE model) and is discussed for best comparison with TBED‐C. *p < 0.05, **p < 0.01, ***p < 0.001.

#### TEDS

Replicating the extended univariate G×E AE model with AE moderation in TEDS supported the presence of increasing nonshared environmental influences on CU traits with increasing anxiety (E1 = 0.87, *p* < 0.001; Table 1; Figure 2). Additionally, we found statistically significant heritability moderation, with decreasing A with increasing anxiety (A1 = −0.22, *p* < 0.01; Figure 2). The direction of the A moderation was consistent across samples, although only significant in TEDS, likely due to increased power to detect small but significant effects. For transparency, we also report results from the full ACE moderation model, which showed slightly better (though not statistically significantly better) fit (Table 1). In this model, the C moderation path was large and negative, and A moderation reversed direction. However, when C moderation was constrained to zero, A moderation became negative (i.e., consistent with the AE model) suggesting that the A moderation sign reversal likely reflects instability introduced by estimating C moderation when baseline C is very small (Purcell, 2002; van der Sluis et al., 2012). E moderation remained significant and consistent in magnitude and direction across models, supporting the robustness of this effect.

The bivariate G×E model confirmed that the etiologic moderation was specific to the unique path (E1 unique = 0.85, *p* < 0.001, E1 shared = −0.16, *p* < 0.001). Sensitivity analyses conducted without regressing out age and sex effects produced equivalent results (Table S4). Finally, a sensitivity analysis with externalizing as the variable of interest (instead of CU traits) indicated no evidence that anxiety moderated genetic or nonshared environmental influences on externalizing (Table S5).

## Discussion

We tested whether levels of anxiety moderated the etiology of CU traits in two large samples of twins from two different countries (USA and UK) and found compelling evidence for moderation in both samples, such that nonshared environmental influences on CU traits increased as twin anxiety increased. Moreover, within the second, larger sample, we found some evidence for decreasing genetic influences on CU traits with increasing anxiety. These findings support theory and clinical evidence suggesting that co‐occurring levels of anxiety identify youth who develop CU traits for different reasons from their peers with CU traits without co‐occurring anxiety (Kimonis, 2023; Viding & Kimonis, 2018). These findings have major implications for theoretical models of CU traits and conduct problems. Furthermore, they have potential implications for intervention, as measuring co‐occurring anxiety may be key to effective personalized treatments for youth with CU traits. A considerable strength of this study is the use of a twin cohort with enrichment for adversity exposure and a preregistered follow‐up analysis in a second, very large sample drawn from a different country, using complementary but not identical assessment measures.

Consistent with previous literature (Moore et al., 2019), we found in both samples that CU traits are moderately to strongly heritable with moderate nonshared environmental influences. Using a genotype‐by‐environment interaction modeling technique that accounts for gene–environment correlation and allows twins to differ on the moderator variable, we found that nonshared environmental influences increased as twin anxiety increased. Notably, the moderation of nonshared environmental influences was consistent across samples and maintained both its direction and significance in the TEDS sample across the full (ACE) and reduced (AE) models. This finding lends support to the contention that secondary CU traits may be a behavioral “phenocopy” of primary CU traits, emerging as an adaptation to early environmental circumstances, such as adversity and trauma (Cecil, McCrory, Barker, Guiney, & Viding, 2017; Kimonis et al., 2013; Viding & Kimonis, 2018), that may differ between co‐twins and, as a result, contribute to the nonshared environmental component of variance in the twin design. In this context, anxiety may serve as a marker of environmental sensitivity, amplifying the impact of nonshared environmental factors. This possibility aligns with meta‐analytic findings that anxiety moderates the association between childhood maltreatment and CU traits, with a stronger association at higher levels of anxiety (Todorov, Devine, & De Brito, 2023).

We also found evidence in the larger TEDS sample that genetic influences may decrease with increasing anxiety. This finding complements recent work showing differences in the heritability of CU traits by environmental factors such as parenting (Henry et al., 2018; Tomlinson et al., 2022). It also offers further support to the theory that primary and secondary variants of CU traits differ in their etiology, and particularly in their heritability, such that primary CU traits, featuring low anxiety, are more strongly heritable (Kimonis, 2023). Varying heritability as a function of anxiety levels could explain some of the heterogeneity that is seen in heritability estimates for CU traits across studies (Moore et al., 2019): It may be that samples in which more individuals happen to have co‐occurring anxiety show lower heritability. Notably, although this finding fits with theory in the field, it stands in contrast to the only other study to address a similar question in a smaller subsample of TEDS and with a different statistical approach, which found no evidence of differential heritability by anxiety for twins who had the highest levels of CU traits (Humayun et al., 2014). In the current analyses, the negative moderation of genetic influences was observed in the AE model and was preserved in the ACE model with AE moderation (i.e., C estimated but not allowed to vary with anxiety), but reversed direction when C moderation was allowed. This pattern suggests that the A moderation effect is directionally stable unless C moderation is modeled. Given that estimates of C in TEDS were very small at baseline, this sign reversal may reflect parameter instability introduced by estimating C moderation (Purcell, 2002; van der Sluis et al., 2012; Verhulst, Prom‐Wormley, Keller, Medland, & Neale, 2019). An alternative, though less likely interpretation is that the very small shared environmental effects themselves may decrease with increasing anxiety, and that constraining C moderation to zero artificially attributes this variance to genetic moderation.

In addition to implications for theories of CU trait development, these theoretical insights have practical implications for treatment approaches. Taken together with current research and theory on primary versus secondary CU traits (Craig et al., 2020; Kimonis, 2023), our findings suggest that providers may want to consider different treatment options for children who present with CU traits and co‐occurring anxiety. Importantly, youth with “secondary” CU traits (i.e., elevated CU traits co‐occurring with high anxiety) often present with greater comorbidity, including internalizing and externalizing problems (Kimonis, 2023; Kimonis, Frick, Cauffman, Goldweber, & Skeem, 2012). Tailored, integrative models of care could therefore first assess anxiety and comorbidity to determine the appropriate intervention pathway. When anxiety is present, children may benefit from emotion regulation skills training, gold‐standard CBT approaches to address anxiety, or dyadic‐relational approaches to enhance parent perspective‐taking and address childhood trauma (Daros et al., 2021; Walkup et al., 2008). In contrast, when co‐occurring anxiety is not present, repeated, intensive, and multicomponent treatments intended specifically to address CU traits, over longer periods of time, may be in order (Pezzoli et al., 2024).

The strengths of this study are underscored by the complementary nature of the two samples: one enriched for adversity exposure, representing modestly‐to‐severely impoverished neighborhoods in the USA, and the other a large cohort representative of UK families. The use of differing but complementary measures of CU traits and anxiety across samples further minimizes the risk that observed moderation effects are simply artifacts of measurement. These strengths noted, our findings should be interpreted in the context of the following limitations. First, we did not have measures of trauma in these samples and therefore could not test whether interaction effects were specific to anxiety emerging from childhood trauma. However, anxiety alone, independent of trauma, has been found to distinguish primary and secondary CU variants in both clinical and community samples (Todorov et al., 2024). From a diagnosis and treatment standpoint, anxiety is also straightforward to assess via well‐established questionnaires. Thus, while trauma likely plays an important role, focusing on anxiety moves the work closer to translation into clinical practice. Second, our measurement of CU traits and anxiety relied exclusively on maternal report. Although parent report is useful for outcomes like CU traits, reliance on a single informant limits our ability to distinguish true child‐level associations from potential rater bias. Multi‐informant designs (e.g., teacher report, observational methods) would strengthen construct validity in future work. Third, analyses were conducted in population‐based samples, and findings may differ in selected clinical or correctional samples of youth with CU traits (Humayun et al., 2014; Moore et al., 2019). Fourth, although CU traits and conduct problems are correlated, we did not covary for conduct problems when modeling CU traits because this approach can be conceptually problematic (i.e., what does the variance in CU traits that does not overlap with CP mean?). CU traits are theorized to distinguish subgroups of children with conduct problems and measures addressing these traits were originally developed for this purpose. Residualizing CU traits on conduct problems would therefore remove the very variance that makes CU traits meaningful in relation to understanding behavioral disturbance and distort the construct under study. Instead, to address the possibility that our findings simply reflected conduct problems more broadly, we conducted a sensitivity analysis using externalizing symptoms as the outcome. Anxiety did not moderate genetic or nonshared environmental influences on externalizing, supporting the specificity of the findings to CU traits. Fifth, this study considered anxiety dimensionally as a continuous moderator. Such an approach is consistent with precedent in the field (Endler & Kocovski, 2001); however, while theory and group‐based modeling tends to divide primary and secondary CU traits into two distinct groups (Craig et al., 2020; Kimonis, 2023), it remains to be clarified what levels of anxiety define “primary” versus “secondary” CU traits. Sixth, samples were predominantly White, which may limit generalizability. While research shows broad cross‐cultural stability in core CU traits, cultural‐specific manifestations have been documented (Deng et al., 2024). Finally, these analyses were conducted in middle childhood. Given that the heritability of CU traits increases across development (Moore et al., 2019), additional investigation is needed to assess whether moderation effects may change across development.

In summary, we found consistent evidence across two independent twin samples that anxiety moderates the etiology of CU traits, such that increasing twin anxiety is associated with increasing nonshared environmental influences. Results in the larger sample indicated that anxiety may be associated with decreasing genetic influences. These findings underscore the importance of assessing co‐occurring anxiety in children with CU traits, both in clinical and research settings, as the presence of anxiety may identify differing etiology of CU traits and the need for distinct therapeutic approaches.

## Ethical considerations

For TBED‐C, parents provided informed consent, and children provided assent in compliance with the policies of the Institutional Review Board of Michigan State University (MSU IRB# 04‐887 approved 12/05/06 and #17‐746R approved 06/23/17). For TEDS, parents provided informed consent in compliance with the policies of the ethics committee for the Institute of Psychiatry, Psychology, and Neuroscience at King's College London (Reference Numbers: PNM/09/10‐104 approved 09/06/2021).

**Key points**

**What's known?**

- Callous–unemotional (CU) traits are associated with more severe and persistent conduct problems in youth, but their etiology may differ depending on co‐occurring anxiety.

**What's new?**

- This study used genotype‐by‐environment twin modeling in two large population‐based cohorts to examine whether anxiety moderates genetic and environmental influences on CU traits.
- Across both samples, higher anxiety was associated with increased nonshared environmental influence on CU traits.

**What's relevant?**

- These findings suggest that assessing co‐occurring anxiety may help identify different developmental pathways to CU traits, which has implications for tailoring interventions in clinical settings.

## Supporting information


**Table S1.** Descriptive statistics.
**Table S2.** Cross‐trait, cross‐twin correlations with confidence intervals for TBED‐C and TEDS samples.
**Table S3.** Model estimates and model fit statistics for univariate models.
**Table S4.** Genotype × Environment Interaction Models for TBED‐C and TEDS Samples using non‐residualized variables.
**Table S5.** Sensitivity Analysis: Genotype × Environment Interaction Model with externalizing problems as primary phenotype.

## Data Availability

TBED‐C data cannot be posted publicly due to the language in the informed consent document at intake. These data can be obtained from Prof. A.S.B. upon reasonable request and with an institutional data use agreement. TEDS data are available upon request (teds.ac.uk/researchers/teds‐data‐access‐policy).
