# Supplementary material for: The nature and nurture of primary and secondary callous–unemotional traits: evidence from two independent twin samples
Source: J Child Psychol Psychiatry. 2026 Jan 5;67(6):988–97. doi: 10.1111/jcpp.70107 (PMC13170624; doi:10.1111/jcpp.70107)
Supplement: Supplementary file 1 — Table S1. Descriptive statistics. Table S2. Cross‐trait, cross‐twin correlations with confidence intervals for TBED‐C and TEDS samples. Table S3. Model estimates and model fit statistics for univariate models. Table S4. Genotype × Environment Interaction Models for TBED‐C and TEDS Samples using non‐residualized variables. Table S5. Sensitivity Analysis: Genotype × Environment Interaction Model with externalizing problems as primary phenotype. [file JCPP-67-988-s001.docx]

**The nature and nurture of primary and secondary callous-unemotional traits:**

**Evidence from two independent twin samples**

**Supporting Information**

Table S1. Descriptive statistics

**TBED-C**

| Variable |  | N | Mean | SD | Median | Min | Max | Skew | Kurtosis | SE |
| --- | --- | --- | --- | --- | --- | --- | --- | --- | --- | --- |
| CU Traits |  | 1,189 | 15.44 | 9.85 | 13.39 | 0.00 | 57.00 | 0.90 | 0.55 | 0.29 |
|  | Females | 587 | 14.51 | 9.26 | 12.27 | 0.00 | 46.51 | 0.89 | 0.28 | 0.38 |
|  | Males | 602 | 16.34 | 10.32 | 14.55 | 0.00 | 57.00 | 0.87 | 0.58 | 0.42 |
| Anxiety |  | 1,192 | 1.59 | 1.93 | 1.00 | 0.00 | 11.00 | 1.52 | 2.37 | 0.06 |
|  | Females | 589 | 1.57 | 1.89 | 1.00 | 0.00 | 10.00 | 1.51 | 2.23 | 0.08 |
|  | Males | 603 | 1.62 | 1.97 | 1.00 | 0.00 | 11.00 | 1.53 | 2.45 | 0.08 |

**TEDS**

| Variable |  | N | Mean | SD | Median | Min | Max | Skew | Kurtosis | SE |
| --- | --- | --- | --- | --- | --- | --- | --- | --- | --- | --- |
| CU Traits |  | 13,486 | 3.05 | 1.99 | 3.00 | 0.00 | 14.00 | 0.66 | 0.42 | 0.02 |
|  | Females | 6,953 | 2.79 | 1.89 | 3.00 | 0.00 | 11 | 0.67 | 0.39 | 0.02 |
|  | Males | 6,533 | 3.33 | 2.06 | 3.00 | 0.00 | 14 | 0.61 | 0.37 | 0.03 |
| Anxiety |  | 13,486 | 10.93 | 6.71 | 10.00 | 0.00 | 46.00 | 0.93 | 0.90 | 0.06 |
|  | Females | 6,953 | 11.39 | 6.71 | 10.00 | 0.00 | 42 | 0.84 | 0.62 | 0.08 |
|  | Males | 6,533 | 10.44 | 6.67 | 9.00 | 0.00 | 46 | 1.04 | 1.29 | 0.08 |

Table S2

*Cross-trait, cross-twin correlations with confidence intervals for TBED-C and TEDS samples*

| Variable | 1 | 2 | 3 | 4 |
| --- | --- | --- | --- | --- |
|  |  |  |  |  |
| 1. CU Traits T1 |  | **0.37**** | -0.02* | 0.06** |
|  |  | [0.35, 0.39] | [-.05, .00] | [0.04, 0.08] |
|  |  |  |  |  |
| 2. CU Traits T2 | **0.65**** |  | 0.06** | -0.02* |
|  | [0.63, 0.66] |  | [0.04, 0.08] | [-0.05, 0.00] |
|  |  |  |  |  |
| 3. Anxiety T1 | -0.01 | 0.06** |  | **0.44**** |
|  | [-0.04, 0.02] | [0.03, 0.09] |  | [0.42, 0.46] |
|  |  |  |  |  |
| 4. Anxiety T2 | 0.06** | -0.01 | **0.69**** |  |
|  | [0.03, 0.09] | [-0.04, 0.02] | [0.68, 0.71] |  |
|  |  |  |  |  |

TBED-C TEDS

| Variable | 1 | 2 | 3 | 4 |
| --- | --- | --- | --- | --- |
|  |  |  |  |  |
| 1. CU Traits T1 |  | **0.20**** | 0.20** | 0.16** |
|  |  | [0.12, 0.26] | [0.13, 0.27] | [0.08, 0.23] |
|  |  |  |  |  |
| 2. CU Traits T2 | **0.46**** |  | 0.16** | 0.20** |
|  | [0.39, 0.53] |  | [0.08, 0.23] | [0.13, 0.27] |
|  |  |  |  |  |
| 3. Anxiety T1 | 0.10* | 0.07 |  | **0.40**** |
|  | [0.01, 0.19] | [-0.02, 0.16] |  | [0.33, 0.46] |
|  |  |  |  |  |
| 4. Anxiety T2 | 0.07 | 0.10* | **0.42**** |  |
|  | [-0.02, 0.16] | [0.01, .19] | [0.34, 0.49] |  |
|  |  |  |  |  |

*Note.* Values in square brackets indicate the 95% confidence interval for each correlation. Correlations for monozygotic twins are included below the diagonal and dizygotic twins above the diagonal. T1 and T2 indicate twin 1 and twin 2. Correlations were generated from a double-entered dataset to eliminate any twin ordering effects. Correlations which represent ICCs for CU traits and anxiety are bolded for clarity. * indicates *p* < .05. ** indicates *p* < .01.

Table S3

*Model estimates and model fit statistics for univariate models*

| TBED-C | | | | | | | | |
| --- | --- | --- | --- | --- | --- | --- | --- | --- |
| Univariate Model | A | C | E |  | AIC | BIC | ssBIC | RMSEA |
| CU traits ACE | 0.51*** | 0.00 | 0.49*** |  | 3311.58 | 3329.15 | 3316.46 | 0.12 |
| **CU traits AE** | **0.51***** |  | **0.49***** |  | **3309.58** | **3322.76** | **3313.24** | **0.11** |
| TEDS | | | | | | | | |
| Univariate Model | A | C | E |  | AIC | BIC | ssBIC | RMSEA |
| CU traits ACE | 0.59*** | 0.07*** | 0.34*** |  | 18688.51 | -183825.2 | 72624.51 | 0.00 |
| CU traits AE | 0.67*** |  | 0.31*** |  | 18698.39 | -183822.8 | 72607.87 | 0.03 |

*Note.* This table depicts estimates and model fit statistics for the univariate twin models, which provide a decomposition of genetic and environmental contributions to variance for Callous-Unemotional (CU) traits. Within TBED-C (N=591 pairs, 228 MZ), the best fitting and more parsimonious univariate model was the AE model, depicted in bold. Within TEDS (N=6,743 pairs, 2,444 MZ), the best fitting and more parsimonious univariate model was the ACE model, depicted in bold. For estimates, * p <0.05, ** p<0.01, *** p<0.001.

Table S4

*Genotype x Environment Interaction Models for TBED-C and TEDS Samples using non-residualized variables*

| TBED-C | | | | | | | | |
| --- | --- | --- | --- | --- | --- | --- | --- | --- |
| Model | **A** | **A1** | **C** | **C1** | **E** | **E1** | **AIC** | **BIC** |
| ACE,  ACE moderation | 0.67***  [0.58, 0.76] | -0.03  [-0.69, 0.63] | -0.16  [-0.55, 0.22] | 0.61  [-0.80, 2.02] | 0.63***  [0.54, 0.71] | 0.55*  [0.06, 1.05] | 3247.83 | 3300.49 |
| **AE,**  **AE moderation** | **0.69*****  **[0.59, 0.80]** | **-0.14**  **[-0.57, 0.30]** |  |  | **0.62*****  **[0.52, 0.72]** | **0.61***  **[0.03, 1.19]** | **3243.92** | **3287.81** |
|  |  |  |  |  |  |  |  |  |
| TEDS | | | | | | | | |
| Model | **A** | **A1** | **C** | **C1** | **E** | **E1** | **AIC** | **BIC** |
| **ACE,**  **ACE moderation** | **0.59*****  **[0.54, 0.65]** | **0.59*****  **[0.42, 0.76]** | **0.75*****  **[0.69, 0.80]** | **-1.78*****  **[-1.97, -1.59]** | **0.39*****  **[0.36, 0.41]** | **0.62*****  **[0.51, 0.72]** | **35718.94** | **35800.74** |
| ACE,  AE moderation | 0.80***  [0.76, 0.84] | -0.28**  [-0.43, -0.13] | 0.34***  [0.28, 0.40] |  | 0.35***  [0.33, 0.38] | 0.90***  [0.80, 1.00] | 35824.67 | 35899.65 |
| AE,  AE moderation | 0.86***  [0.83, 0.89] | -0.20**  [-0.32, -0.08] |  |  | 0.35***  [0.33, 0.38] | 0.84***  [0.74, 0.94] | 35841.50 | 35909.67 |
|  |  |  |  |  |  |  |  |  |

*Note.* This table depicts unstandardized estimates and model fit statistics for the extended univariate genotype x environment (GxE) interaction models without regressing out age and sex effects. N=595 pairs (230 monozygotic) for TBED-C, N=6,743 pairs (2,444 MZ) for TEDS. The first model listed for each moderator is the full ACE model of callous-unemotional traits by anxiety, with linear moderation (A1, C1, E1) allowed on A, C, and E terms. The best-fitting models are indicated in bold. In the replication sample, while the ACE moderation model fit the data best, the AE moderation model also demonstrated a strong fit and is discussed for best comparison with TBED-C. * p <0.05, ** p<0.01, *** p<0.001

Table S5

*Sensitivity Analysis: Genotype x Environment Interaction Model with externalizing problems as primary phenotype*

| TBED-C | | | | | | | | |  |
| --- | --- | --- | --- | --- | --- | --- | --- | --- | --- |
| Model | **A** | **A1** | **C** | **C1** | **E** | **E1** | **AIC** | **BIC** | |
| AE,  AE moderation | 0.68***  [0.59, 0.77] | -0.07  [-0.74, 0.60] |  |  | 0.60***  [0.52, 0.67] | -0.02  [-0.57, 0.53] | 3006.94 | 3050.82 | |
|  |  |  |  |  |  |  |  |  | |
|  |  |  |  |  |  |  |  |  | |

*Note.* This table depicts unstandardized estimates and model fit statistics for extended univariate genotype x environment (GxE) interaction models with externalizing problems as the phenotype of interest (rather than callous-unemotional traits). N=595 pairs (230 monozygotic). In contrast to the CU trait models, there was no evidence of anxiety-related moderation of nonshared environmental influences on externalizing problems. * p <0.05, ** p<0.01, *** p<0.001
